# Supplementary material for: Postoperative complication management: How do large language models measure up to human expertise?
Source: PLOS Digit Health. 2025 Aug 1;4(8):e0000933. doi: 10.1371/journal.pdig.0000933 (PMC12316209; doi:10.1371/journal.pdig.0000933)
Supplement: S1 Table — (DOCX) [file pdig.0000933.s001.docx]

**S1 Table.** Postoperative patient cases.

| **Case** | **Case Description** |
| --- | --- |
| Case 1: Anastomotic leakage | Gender: Male. Age: 62 years. Postoperative Day: 4. Surgery: Ileostomy relocation after rectum resection for rectal carcinoma. Abdomen: Bowel sounds in all four quadrants, strong guarding and pain throughout the abdomen, skin is unremarkable, Lungs: no pathologic findings, Pulse: 180/min. Nasogastric tube is in place and yielding small amount of bile. Patient has already received pain medication and infusion as part of a fluid therapy. Medications: Anticoagulation, antihypertensives. Currently in place foreign material: Vascular access, Indwelling catheter. |
| Case 2: Stroke | Postoperative Patient Case. Gender: Male. Age: 83 years. Postoperative Day: 4. Surgery: Left hemicolectomy with anastomosis of continuity for rectal carcinoma. The patient suddenly developed a hemiparesis of the right side of the body, initially noticed during lunch when he dropped his fork and presented with motor aphasia. The nurse checked his vital functions and connected a monitor (vital parameters unremarkable), and an i.v. access was present. The only pre-existing condition was arterial hypertension, treated with an ACE inhibitor and a beta-blocker. The following blood tests have already been taken from the patient: CBC, CRP, blood sugar, electrolytes, lactate, creatinine, urea, GFR, bilirubin, ALT, AST, y-GT, troponin, CK, TSH, INR, pTT, ethanol. The values have not changed significantly from the pre-values and are monitored continuously.. Medications: ACE inhibitor and beta-blocker Current foreign materials in place: Vascular access. Foreign material present: Peripheral venous catheter. |
| Case 3: Postoperative pancreatic fistula (POPF) | Gender: Female. Age: 65 years. Postoperative Day: 4. Operation: Distal Pancreatectomy for Pancreatic Carcinoma in the Pancreatic Tail. You are called to a 65-year-old patient who has shown elevated temperature (in the morning: 39.4°C) for the first time today. The patient also presents in a reduced general condition, shows abdominal guarding in the upper abdomen and reports abdominal pain radiating to the back. Blood pressure: 105/70, Pulse 130. The surgical wound looks unremarkable. Costovertebral angle tenderness is present. The patient no longer has a nasogastric tube, the drainage yields turbid fluid. This morning a venous blood test was done and blood cultures were taken and sent to the microbiology. While the blood cultures have arrived, the laboratory samples have not reached the laboratory and are not traceable. Medications: Anticoagulation, Antihypertensives. Current foreign Material: Peripheral venous catheter, Drainage. |
| Case 4: Mechanical ileus | Gender: Male. Age: 87 years. Postoperative Day: 2. Surgery: Right hemicolectomy (long Hartmann's = right hemicolectomy with creation of a terminal ileostomy and blind closure of the remaining colon) for colonic ischemia. At the afternoon visit, the patient presents today with abdominal pain and nausea and has already vomited twice during the day. The abdomen is distended, the ostomy bag is empty. The patient is currently taking pureed food. The surgical wound is non irritated and the drainage is serous. The nursing staff has already administered metamizole (pain medication) and granisetron (for nausea) from the demand medication. Medications: Metamizole, Granisetron. Currently present foreign materials: Peripheral venous catheter, Stoma with ostomy bag, drainage, long-term urinary catheter. |
| Case 5: COVID-19 / wound infection | Gender: Female. Age: 68 years. Postoperative day: 14. Surgery: Rectal excision for recurrent anal carcinoma. The GP has referred the patient again due to increasing CRP, perineal wound infection with purulent secretion, some fever, headaches and circulatory problems. A wound swab was already taken when the sutures were removed (a few days ago) and antiseptic lavage was started. Otherwise, the patient is without pathological findings, and the stoma is functioning properly. A rapid COVID test was conducted in the emergency room, which was negative, and the result of the PCR is still awaited. The patient has not yet been clinically examined. Medications: antihypertensives, antidiabetics. Foreign material currently in place: stoma with ostomy bag. |
| Case 6: Sentinel bleeding | Gender: Female. Age: 71. Postoperative day: 7. Operation: Whipple procedure for pancreatic head carcinoma. The patient's drainage fluid is tinged with blood. Known pancreatic fistula (elevated amylase levels in the drainage secretion, no clinical symptoms), otherwise uneventful course. She also presents in a reduced general condition with circulatory dysfunction and diffuse abdominal pain. The surgical scar looks normal. The patient also has atrial fibrillation as a concomitant condition and has been taking Marcumar (vitamin K antagonist) again since the day before as perioperative bridging.  The venous blood test showed a decrease in Hb from 10.5 g/dl to 7.5 g/dl. Medications: Vitamin K antagonist. Currently implanted foreign material: Peripheral venous catheter, drainage, urinary catheter, metal staples. |
