# Supplementary material for: Postoperative complication management: How do large language models measure up to human expertise?
Source: PLOS Digit Health. 2025 Aug 1;4(8):e0000933. doi: 10.1371/journal.pdig.0000933 (PMC12316209; doi:10.1371/journal.pdig.0000933)
Supplement: S2 Table — (DOCX) [file pdig.0000933.s002.docx]

**S2 Table.** Ideal diagnostic and therapeutic pathways for all cases of postoperative complications according to German and European treatment guidelines as well as current scientific evidence (Wente et al., 2009; Bassi et al., 2017; Kulu et al., 2013; Welsch et al., 2016).

| **Case** | **Diagnostic and therapeutic measures** |
| --- | --- |
| Anastomotic leakage | Diagnostics:   - Abdominal X-ray to evaluate for any obstruction or ileus. - Electrocardiogram to evaluate for any cardiac arrhythmias. - Blood work to assess electrolyte levels, liver and kidney function. - Urinalysis to assess for any infection. - Stool culture to assess for any infection.   Therapy:   - Provide adequate pain control with analgesia. - Provide adequate hydration and electrolyte balance with intravenous fluids. - Monitor for any signs of infection, such as fever and white blood cell count. - Monitor for any signs of bleeding, such as hemoglobin/hematocrit levels. - Monitor for any signs of anemia, such as hemoglobin/hematocrit levels. - Monitor for any signs of electrolyte imbalance, such as sodium, potassium and magnesium levels. - Monitor for any signs of infection, such as fever and leukocytosis. - Monitor for any signs of dehydration, such as increased hematocrit. - Monitor for any signs of nutritional deficiency, such as albumin levels. |
| Stroke | Diagnostics:   - CT-Scan of the head and neck to rule out a stroke or other neurological disorder. - Echocardiogram to assess for cardiac involvement. - EEG to assess for brain activity. - MRI to check for any brain lesions or other abnormalities.   Therapy:   - Administer oxygen therapy. - Administer anti-hypertensives and beta-blockers as prescribed. - Administer anticoagulants as prescribed. - Administer anti-epileptic drugs as prescribed. - Administer anti-inflammatory medications to reduce swelling and inflammation. - Administer physical, occupational, and speech therapies as needed. - Monitor vital signs and electrolytes regularly. - Monitor neurological and behavioral status regularly. |
| POPF | Diagnostics:   - Complete blood count (CBC). - Blood culture. - Imaging tests such as X-rays, CT scans, or ultrasound to investigate the cause or the pain. - Urinalysis.   Therapy:   - Administer an appropriate antibiotic based on the infection risk, such as broad-spectrum antibiotic. - Administer appropriate pain medications. - Maintain hydration and electrolyte balance through IV fluids, if necessary. - Monitor vital signs regularly and adjust medications accordingly. - Monitor drainage output and change drainage bags, if necessary. - For wound care, keep the wound clean and dry, and check for signs of infection. - Monitor for signs of deep vein thrombosis and pulmonary embolism. |
| Mechanical ileus | Diagnostics:   - Abdominal x-ray or ultrasound to assess bowel distension and any possible obstruction. - Blood tests to check for signs of infection and electrolyte imbalances. - Stool culture to check for infection.   Therapy:   - IV fluids to help manage dehydration. - Antibiotics if an infection is detected. - Prokinetic agents to help restore bowel motility. - Antiemetics to help with nausea and vomiting. - Pain medication if needed. - Refeeding with a low-residue diet to help reduce symptoms of obstruction. |
| COVID-19 / wound infection | Diagnostics:   - Complete blood count (CBC). - C-reactive protein (CRP). - Wound swab for bacterial culture. - Urinalysis. - PCR for COVID.   Therapy:   - Antibiotic therapy targeted to the bacteria identified in the wound swab. - Pain management. - Stoma care to prevent further infection. - Close monitoring of vital signs. - Close monitoring of wound healing. - Close monitoring of electrolytes. - Adequate nutrition and hydration. - Physical therapy to prevent contractures and muscle wasting. |
| Sentinel bleeding | Diagnostics:   - Complete blood count (CBC) test to evaluate tor anemia and other abnormalities. - Biochemical tests such as amylase, lipase, and electrolytes to assess for possible pancreatic fistula. - CT scan of the abdomen and pelvis to evaluate for any complications from the surgery, such as abscesses, fluid collections, or fistulas. - Abdominal ultrasound to further assess for any complications from the surgery.   Therapy:   - Administer intravenous fluids to support circulatory function and to correct electrolyte imbalances. - Administer packed red blood cells (PRBCs) or other blood product transfusions to correct anemia. - Administer antibiotics if there is evidence of an infection. - Control pain with appropriate medications (e.g., opioids). - Monitor patient's vital signs, laboratory values, and other relevant parameters closely. - Monitor for any signs of pancreatic fistula, such as increased amylase levels in drainage secretions, and initiate appropriate treatment if necessary. - Optimize anticoagulation therapy as needed. |
