# Supplementary material for: Postoperative complication management: How do large language models measure up to human expertise?
Source: PLOS Digit Health. 2025 Aug 1;4(8):e0000933. doi: 10.1371/journal.pdig.0000933 (PMC12316209; doi:10.1371/journal.pdig.0000933)
Supplement: S3 Table — The table outlines the number of correct triage assessments. Specification of the organ system was mandatory, while specification of complication mechanism and exact complication were voluntary. Data were published previously in Schwarzkopf et al., JMIR Serious Games (2023). (DOCX) [file pdig.0000933.s003.docx]

**S3 Table.** **Evaluation of the suspected diagnosis by suspected affected organ system, suspected complication mechanism, and suspected exact complication across all postoperative cases, disaggregated by competency levels of human caregivers.** The table outlines the number of correct triage assessments. Specification of the organ system was mandatory, while specification of complication mechanism and exact complication were voluntary. Data were published previously in Schwarzkopf et al., JMIR Serious Games (2023).

|  | Organ system | | | | |
| --- | --- | --- | --- | --- | --- |
|  | Experts | Board-certified surgeons | Surgical residents | Medical students | Non-medical staff |
| Correct | 18/18  (100%) | 5/5  (100%) | 11/14  (79%) | 70/74  (95%) | 17/20  (85%) |
| Incorrect | 0/18  (0%) | 0/5  (0%) | 3/14  (21%) | 4/74  (5%) | 3/20  (15%) |
| NA | 0/18  (0%) | 0/5  (0%) | 0/14  (0%) | 0/74  (0%) | 0/20  (0%) |
|  | | | | | |
|  | Complication mechanism | | | | |
|  | Experts | Board-certified surgeons | Surgical residents | Medical students | Non-medical staff |
| Correct | 14/18  (78%) | 4/5  (80%) | 12/14  (86%) | 34/74  (46%) | 13/20  (65%) |
| Incorrect | 0/18  (0%) | 0/5  (0%) | 0/14  (0%) | 7/74  (9%) | 4/20  (20%) |
| NA | 4/18  (22%) | 1/5  (20%) | 2/14  (14%) | 33/74  (45%) | 3/20  (15%) |
|  | | | | | |
|  | Exact complication | | | | |
|  | Experts | Board-certified surgeons | Surgical residents | Medical students | Non-medical staff |
| Correct | 6/18  (33%) | 2/5  (40%) | 5/14  (36%) | 11/74  (15%) | 5/20  (25%) |
| Incorrect | 1/18  (6%) | 0/5  (0%) | 1/14  (7%) | 1/74  (1%) | 1/20  (5%) |
| NA | 11/18  (61%) | 3/5  (60%) | 8/14  (57%) | 62/74  (84%) | 14/20  (70%) |
