# Supplementary material for: Postoperative complication management: How do large language models measure up to human expertise?
Source: PLOS Digit Health. 2025 Aug 1;4(8):e0000933. doi: 10.1371/journal.pdig.0000933 (PMC12316209; doi:10.1371/journal.pdig.0000933)
Supplement: S4 Table — (DOCX) [file pdig.0000933.s004.docx]

**S4 Table.** Evaluation of counterfactual changes in patient parameters and LLM temperature using GPT-4o for emergency assessment and identification of underlying complications in the anastomotic leakage case.

| **Changed Parameter** | **Emergency** | **Spectrum** | **Specification** | **Initial Diagnosis** |
| --- | --- | --- | --- | --- |
| **Correct answer** | No | Gastrointestinal | Infection complication | Anastomotic leak |
| **Age [years]** |  |  |  |  |
| 72 (+10) | Yes | Gastrointestinal | Infection complication | Anastomotic leak |
| 65 (+1) | Yes | Gastrointestinal | Infection complication | Anastomotic leak |
| 64 (original) | Yes | Gastrointestinal | Infection complication | Anastomotic leak |
| 63 (-1) | Yes | Gastrointestinal | Infection complication | Anastomotic leak |
| 52 (-10) | Yes | Gastrointestinal | Infection complication | Anastomotic leak |
| **POD [days]** |  |  |  |  |
| 1 (-3) | Yes | Gastrointestinal | Infection complication | Anastomotic leak |
| 4 (original) | Yes | Gastrointestinal | Infection complication | Anastomotic leak |
| 7 (+3) | Yes | Gastrointestinal | Infection complication | Anastomotic leak |
| **BP [mmHg]** |  |  |  |  |
| 200 (+20) | Yes | Gastrointestinal | Infection complication | Anastomotic leak |
| 190 (+10) | Yes | Gastrointestinal | Infection complication | Anastomotic leak |
| 180 (original) | Yes | Gastrointestinal | Infection complication | Anastomotic leak |
| 170 (-10) | Yes | Gastrointestinal | Infection complication | Anastomotic leak |
| 160 (-20) | Yes | Gastrointestinal | Infection complication | Anastomotic leak |
| **Body Temperature [°C]** |  |  |  |  |
| +3 | Yes | Gastrointestinal | Infection complication | Anastomotic leak |
| +1 | Yes | Gastrointestinal | Infection complication | Anastomotic leak |
| Original | Yes | Gastrointestinal | Infection complication | Anastomotic leak |
| -1 | Yes | Gastrointestinal | Infection complication | Anastomotic leak |
| -3 | Yes | Gastrointestinal | Infection complication | Anastomotic leak |
| **Chat interface temperature** |  |  |  |  |
| 0.1 | Yes | Gastrointestinal | Infection complication | Anastomotic leak |
| 0.5 | Yes | Gastrointestinal | Infection complication | Anastomotic leak |
| 0.9 | Yes | Gastrointestinal | Infection complication | Anastomotic leak |
