# Supplementary material for: Postoperative complication management: How do large language models measure up to human expertise?
Source: PLOS Digit Health. 2025 Aug 1;4(8):e0000933. doi: 10.1371/journal.pdig.0000933 (PMC12316209; doi:10.1371/journal.pdig.0000933)
Supplement: Attachment — Submitted filename: Rebuttal Letter_LLM-Comp_PLOS_R1.pdf [file pdig.0000933.s007.pdf]

Detailed Response to Reviewers

Editorial Comments:

Thank you for submitting your manuscript to PLOS Digital Health. After careful consideration, we feel that it has merit but does not fully meet PLOS Digital Health's publication criteria as it currently stands. Therefore, we invite you to submit a revised version of the manuscript that addresses the points raised during the review process. Please pay particular attention to the comments of Reviewer #3 and ensure that your research meets the highest reproducibility standards (e.g. comment about prompting strategy). **[E1]**

Dear Professor Celi, dear PLOS Digital Health Editorial Team,

Thank you for considering our original manuscript entitled "Postoperative Complication Management: How Do Large Language Models Measure Up to Human Expertise?" for publication in PLOS Digital Health and for the opportunity to resubmit a revised manuscript.

We would like to thank you and the reviewers for the recommendations and the very helpful critique. We have addressed the reviewers' suggestions in our attached point-by-point response and revised the manuscript accordingly. All changes to the manuscript have been highlighted using tracked changes. Specifically, we made the following major changes:

- *We added a detailed overview to specify the response pattern to the postoperative cases by study participants (Fig. 3A - 3F, Supplementary Material 2).*
- *We conducted further testing for counterfactual modifications to assess model consistency (Supplementary Material 3 and 4).*

In addition, we have made the suggested changes to improve the clarity of the manuscript and feel that these changes have considerably improved the quality and scientific value of our work. We would be pleased if the revised manuscript meets the criteria for publication in PLOS Digital Health and look forward to your feedback.

Reviewer #1:

The manuscript addresses a timely and significant topic by assessing the role of large language models (LLMs) in postoperative complication management, an area with substantial potential for clinical impact. The inclusion of multiple LLMs (GPT-3, GPT-4, and Gemini) and comparisons with human caregiver performance provides a thorough evaluation of their capabilities and limitations. The study employs a well-structured methodology using realistic cases to systematically assess triage, diagnosis, and management, enhancing its rigor and clarity. Furthermore, the adherence to ethical standards, including IRB approval and participant consent, adds credibility and robustness to the research. **[R1.1]**

> *We sincerely thank the reviewer for their assessment and the valuable comments, which we have addressed in the revision. We believe that these changes have enhanced the clarity and relevance of our work.*

Despite its strengths, the paper has room for improvement. While it highlights the overestimation of urgency by GPT models, a deeper discussion on how this might impact clinical workflows would enhance the implications section. For example, could this bias result in unnecessary resource allocation or potentially improve patient safety by erring on the side of caution? **[R1.2]**

> *We thank the reviewer for this comment and have addressed this point in the revised Discussion section of our manuscript:*
>
> *"While this overestimation might reduce missing emergencies and increase patient safety, it poses a further risk of constraining physicians and resources. Specifically, overestimating a patient's condition as an emergency limits physicians' abilities to allocate treatment appropriately, decreasing their time for severely ill patients."*

Additionally, while the paper notes advancements in GPT models, a concise analysis of the factors driving these improvements, such as enhanced training data, architectural refinements, or more detailed prompt engineering, would provide valuable context. **[R1.3]**

> *We thank the reviewer for pointing out this issue. We addressed this point in the revised Discussion as follows:*
>
> *"Major improvements in the development of GPT-3 to GPT-4 stem from the increased parameters from 175 billion to 1.8 trillion and the ability to process multimodal input."*

Lastly, the discussion acknowledges the need for prospective evaluation but could further elaborate on practical challenges in integrating LLMs into real-world clinical settings, including issues related to model deployment, clinician training, and data privacy. **[R1.4]**

> *We addressed the reviewer's comment and added specifications for the improvements between models in the text as follows:*
>
> *"The clinical implementation of LLMs is often challenged by their integration into hospitals' electronic health records and patient management systems, requiring specific adaptations and refinements. Additionally, two further issues are anticipated regarding this integration. Firstly, intensive user training will be necessary to ensure that it does not create additional burdens in daily work but is perceived as a value-added tool by users. To achieve this, it would be beneficial to include LLMs as part of medical curricula and professional education to educate medical professionals about potential benefits and risks associated with their clinical deployment. Second, sharing highly sensitive healthcare data, even if anonymized, with LLMs, can lead to data privacy concerns that must be adequately addressed. Patients should be informed about the risks and benefits associated with the use of LLMs, and they should have the option to object to the use of LLMs concerning their health data."*

Reviewer #2:

This manuscript is well-written, and evaluates the performance of 3 LLMS in evaluating postop complications. A couple minor comments and suggestions: **[R2.1]**

We sincerely appreciate your assessment and the valuable feedback, which we have considered and that greatly improves the revised manuscript.

1) The competency level of the "human caregivers" are very likely to be heterogenous, given that it spanned medical students to board certified surgeons. Would it be possible to conduct disaggregated analysis comparing the performance of the different subgroups against the LLMs? This might inform us as to whether LLMs are most useful to people at a certain level of training. **[R2.2]**

We thank Reviewer 2 for highlighting this relevant issue. Accordingly, we have added the data from the different subgroups of human caregivers to Table 1, Figure 3, and Supplementary Material 2. The modified text now reads as follows:

*"*With regard to the identification of the exact complication, we conducted a disaggregated analysis of the performance of the human caregiver subgroups across all six postoperative complication cases (**Fig. 3, Supplementary Material 2**). We observed heterogeneous classification patterns among the various healthcare professional subgroups, with performance improvements with increasing professional experience. GPT-4 generally demonstrated performance comparable to or surpassing that of medical students. Surgeons at all experience levels consistently outperformed GPT-4 in correctly identifying the suspected diagnosis. Both GPT-3 and Gemini depicted performance levels that were, on average, inferior to those of medical professionals of all levels.*"*

Moreover, it strikes me that the difficulty of the 6 cases are different (for example, stroke was very straightforward, but anastomotic leak with the strong guarding was not). Therefore, this reviewer suggests including in Table 1 what kind of human caregivers answered which of the cases to better provide context to the results. **[R2.3]**

Thank you very much for your comment. We have divided the human cohort based on professional group and level of education. The corresponding results of the subgroups have been included in Table 1 and Supplementary Material 2, as outlined in **[R2.2]***. In addition, we visualized the performance of different subgroups of human caregivers in Fig. 3A - 3F.*

In addition, please further describe your human cohort. Please define "surgical experts". Please describe whether the human caregivers were all from a certain university (if so, this should be mentioned in the limitations), what sampling design was used, and whether there was any power analysis done. **[R2.4]**

Thank you very much for bringing up this point. We would like to clarify that the human case evaluation had been carried out in a previous study with distinct limitations. We have now added further information to the Methods section. Senior physicians (i.e., consultant level and above) were classified as experts. We have added these details to the Methods section of our revised manuscript:

"Human management was evaluated as described previously. [23] In brief, a cohort of 131 human caregivers with varying experience in abdominal surgery independently assessed the realistic patient cases. The cohort of human caregivers reflected the whole spectrum of healthcare professionals and consisted mainly of medical students (74/131, 56%), general surgery residents and board-certified surgeons (19/131, 15%), and surgical experts (senior and chief physicians) (18/131, 14%). In addition to these participants, nursing staff and other clinical staff with patient contact were included (20/131, 15%). The participants were recruited from the entire German-speaking region, particularly from the medical and nursing staff of the Carl Gustav Carus University Hospital in Dresden and the students of the Carl Gustav Carus Medical Faculty in Dresden. Following a brief case presentation, participants were asked to triage

the virtual patient and make an initial suspected diagnosis. The distribution of cases was as follows: anastomotic leakage (n = 60), stroke (n = 19), POPF (n = 14), mechanical ileus (n = 10), COVID-19 / wound infection (n = 10), and sentinel bleeding (n = 18). As a limitation of the underlying human case evaluation study, no specific sample size considerations or power analyses were conducted. In addition, participants selected cases using a drop-down function, where anastomotic insufficiency was the first case and therefore selected by most participants. ***Supplementary Material 2*** *displays the diagnostic performance of human caregivers of various professional levels in relation to the affected organ system, the complication mechanism, and the exact complication, as published previously. [23]"*

Is there a reason anastomotic leakage was the case presented to human caregivers much more than others? **[R2.5]**

Thank you for your question. As noted in our response to **[R2.4]**, *the data were generated in an open, observational study, in which the human participants were free to select one or multiple cases to address from a drop-down menu. There was no randomization among the six cases, which is a limitation, as this unfortunately led to the formation of uneven groups. The case regarding anastomotic insufficiency was both the first case in the drop-down menu and represents a very common scenario in surgical in-patient settings, both of which we believe contributed to this case being addressed more frequently than the other cases.*

Please clarify line 225-227. In comparison to human caregivers, we observed significant performance differences for the tested LLMs (humans: 89.8% correctly rated vs. GPT-3: 97.7% correctly rated vs. GPT-4: 100% correctly rated vs. Gemini: 51.7%, p < 0.01). --> clarify in what outcome this was? which among the comparisons was p <0.01? This is confusing because the next sentence seems to contradict it: While GPT-3 and GPT-4 were not 228 significantly different from human caregivers (humans: 89.8% correctly rated vs. GPT-3: 229 97.7% correctly rated vs. GPT-4: 100% correctly rated, p = 0.34), Gemini showed a 230 significantly inferior performance (humans: 89.8% correctly rated vs. Gemini: 51.7%, p < 0.01). **[R2.6]**

We thank Reviewer 2 for pointing out this unclarity. We have clarified the respective paragraph:

"In comparison to human caregivers, we observed significant performance differences in the identification of the pathophysiological complication mechanism for Gemini (humans: 89.8% correctly rated vs. Gemini: 51.7%, p < 0.01). GPT-3 and GPT-4 did not perform significantly differently to human caregivers (humans: 89.8% correctly rated vs. GPT-3: 97.7% correctly rated, p = 0.29; humans: 89.8% correctly rated vs. GPT-4: 100% correctly rated, p = 0.11)."

Finally, please provide additional details on how the reviewers evaluated the responses to the cases. For instance, in table 1 for COVID 19 GPT-4 got a score of 3/10-- what does this entail? **[R2.7]**

Thank you for your comment and inquiry. We have adjusted the table accordingly. In the first row of the table, the six cases were classified as either emergencies or non-emergencies. Only the postoperative pancreatic fistula and stroke are considered emergencies, i.e., scenarios that require immediate attention. The subsequent rows indicate whether the human cohort or the LLMs made a correct assessment of "emergency" or "no emergency."

As outlined in the Methods, for the evaluation of triage and the initial suspected diagnosis, each case was processed ten times by each tested LLM to evaluate the variability of LLM outputs. The percentages reflect the proportion of correct responses.

Reviewer #3:

This article compares three language models to a pool of human experts on the tasks of postoperative complication management.

Methodological comments: To better judge the validity and generality of findings, it would have been helpful to learn more about the exact prompting strategies that were tried out here. Some examples that I suspect the authors may have employed, but were not described are few-shot demonstrations, CoT, templating and the likes. **[R3.1]**

We thank the reviewer for their thorough review, helpful suggestions, and comments. We adjusted the Methods section accordingly, including further information about the prompts used. We only used a zero-shot approach with the prompt present at the end of each presented patient case. We specified this aspect in the methods section and included all prompts that were used:

"Each LLM analyzed each case using a three-step testing process:

1. ***Triage**: First, LLMs were prompted to determine if the case represents an emergency. An emergency was defined as a sudden event posing an immediate threat to the patient's life and health, characterized by threatened, disturbed, or failed vital functions due to injury or acute illness. [28] Based on this definition, stroke and POPF were classified as emergencies, whereas sentinel bleeding, anastomotic leakage, mechanical ileus and COVID-19 / wound infection were not considered emergencies.*

   Prompt: "Is this patient case an emergency?"

2. ***Identification of the complication**: This included the identification of the organ system involved (cardiac, respiratory, neurologic, gastrointestinal, renal, or other organ system), the mechanism of the complication (infection, bleeding, or other complication mechanism), and the specific complication.*

   Prompt: "Which spectrum do you suspect the postoperative complication to be in? Options are: Cardiac spectrum, Respiratory spectrum, Neurologic spectrum, Gastrointestinal spectrum, Renal spectrum, Other spectrum.

   You may optionally specify the suspected diagnosis. Please select between: Infection complication, Bleeding complication, Other complication."

   Furthermore, we tested LLMs' diagnostic potential directly by using the prompt "What is the initial suspected diagnosis?".

3. ***Description of appropriate management**: Last, LLMs were prompted to outline the diagnostic and therapeutic measures to manage the identified complication.*

   Prompt: "What diagnostic and therapeutic measures would you order as part of the acute management?"

Similarly, I am curious how exactly the guidelines were provided to the models. I could imagine fine-tuning, RAG, knowledge bases, soft prompts, or memory-augmented networks but am left to speculate. More clarity on this aspect of the study would be helpful. **[R3.2]**

We thank the reviewer for this question. We did not use a RAG approach since we aimed to investigate the capabilities of general public LLMs with no further training. Such use applies to the user approach of many clinicians and patients unfamiliar with more advanced techniques. We added a phrase in the text as follows:

"Prompting was performed without additional augmented retrieval generation (RAG) or in context learning to test a zero-shot approach."

We acknowledge that this basic strategy represents a major limitation of our work and have added a discussion of this limitation to the revised version of our manuscript:

“The use of open-source models could facilitate collaborative development, allowing for extensive validation and customization to specific medical domains. [22] Additionally, combining LLMs with retrieval-augmented generation (RAG) techniques could enhance contextual understanding and accuracy by leveraging external knowledge bases. [21]”

More information on the simulated patient scenario would be interesting. In particular, how long post-op are these artificial patients supposed to be? If they are recent ops, they will likely have rich sensor information available (e.g., heart rates, blood pressures, hepatology, etc.) that would be very effective inputs to the model beyond mere text. **[R3.3]**

We sincerely thank you for your comment. The section "Conceptualization of patient cases" briefly outlines how the patient cases were structured. Although the participants initially received only limited information about the fictitious postoperative patient, they were able to obtain further information, such as laboratory parameters and CT scans or similar data, during the course of the survey by “requesting” meaningful diagnostic measures.

We have added more details based on your suggestion and refer to the full description of the human case review in our previously published work (Schwarzkopf et al., JMIR Serious Games, 2023).

“The simulated patients were two to a maximum of 14 days post their primary surgery. The initial case description gave limited information about the patient, as would be realistic in a clinical management scenario for a previously unseen patient, prior to initiating appropriate diagnostics, such as laboratory or imaging examinations, and starting corresponding therapeutic measures. [23]”

More than stability across re-runs (which can be easily fixed via temperature etc.) I would have been interested to see stability across trivial counterfactual modifications (age one year older, temperature 0.1 degree higher, etc.) of the case to assess whether functionally similar patients receive similar suggestions. **[R3.4]**

We thank the reviewer for this great suggestion and have conducted a preliminary evaluation of counterfactual changes to patient parameters and LLM temperature for the anastomotic leakage case. Here, changes in age, POD, blood pressure, and LLM temperature did not alter the performance of GPT-4o. With regard to the proposed treatment pathway, we observed slight performance variations of GPT-4o with regard to the output of dangerous, inconsistent, or nonsensical measures, or the absence of important measures, yet without a clear pattern related to age, postoperative day, blood pressure, or LLM temperature.

We have added these new results to the revised manuscript and thank the reviewer for this suggestion.

Minor comment: The list of abbreviations includes “serious games” which seems unrelated from the content of the paper and might be a copy/paste artifact from the same authors’ previous submission [23]. **[R3.5]**

Thank you for pointing this error out. We have removed the abbreviation from the directory.

Again, we hope to have addressed the editor's and the reviewers' points of criticism to their satisfaction and would be very pleased if the revised manuscript meets the criteria for publication in *PLOS Digital Health*. We once more thank you very much in advance for your time and re-consideration of this manuscript for publication.
