# Supplementary material for: Postoperative complication management: How do large language models measure up to human expertise?
Source: PLOS Digit Health. 2025 Aug 1;4(8):e0000933. doi: 10.1371/journal.pdig.0000933 (PMC12316209; doi:10.1371/journal.pdig.0000933)
Supplement: Attachment — Submitted filename: PbP Response_LLM-Comp_PLOS_R2.pdf [file pdig.0000933.s008.pdf]

Detailed Response to Reviewers

Editorial Comments:

Thank you for submitting your manuscript to PLOS Digital Health. After careful consideration, we feel that it has merit but does not fully meet PLOS Digital Health's publication criteria as it currently stands. Therefore, we invite you to submit a revised version of the manuscript that addresses the points raised during the review process.

Dear Professor Berens, dear Professor Celi, dear PLOS Digital Health Editorial Team,

Thank you for considering our original manuscript entitled "Postoperative Complication Management: How Do Large Language Models Measure Up to Human Expertise?" for publication in PLOS Digital Health and for giving us the opportunity to resubmit a revised manuscript.
We would like to thank you and the reviewers for the recommendations and constructive critiques. We have addressed the reviewer's remaining suggestions in our attached point-by-point response and revised the manuscript accordingly. All changes to the manuscript have been highlighted using track changes. Specifically, we have made the following changes:

- *We performed additional testing of body temperature change for counterfactual modifications to assess model consistency (Supplementary Material 3 and 4).*
- *We adapted wording throughout the manuscript.*

We would be pleased if the revised manuscript meets the criteria for publication in PLOS Digital health and look forward to your feedback.

Sincerely,
Dr. Fiona Kolbinger

Reviewer #3:

Comment 1: I would like to thank the authors for their clarifications. A purely zero-shot prompting approach without any form of role or system prompt (e.g., the commonly used "you are a helpful assistant") is rather naive and not reflective of the true fidelity these models can reach if used correctly. What is reported here can only be considered a lower bound to model performance. I suggest adding this as a limitation to the study.

We thank the reviewer for this comment. We acknowledge that our zero-shot approach is only suited to investigate baseline performance and agree that this does not fully leverage the potential of LLMs. We added this aspect to the limitations section as follows (line 423): "In our study we deployed a simplified, zero-shot prompting approach, to investigate baseline LLM capabilities. We acknowledge that this does not leverage the full potential of LLMs, which are able to perform significantly better with structured and more elaborate prompts."

Comment 2: Thanks for clarifying that no RAG etc. was used. From the answer, I guess that the guidelines were not made available to the model, and that, instead, we are testing to which degree guideline-conforming behavior is captured by the models' internal parameters.

Thank you. We did not provide guidelines and tested only basic model capabilities in a zero-shot approach. The models were not specifically provided with guidelines during the testing process. Guideline adherence was therefore based on the internal parameters and underlying training data of each model. We clarified this in the text as follows (line 131): "Therefore, models were only tested upon their baseline training data."

Comment 3: The counterfactual analysis is interesting. I might have been cryptic in my comment when talking about temperatures in two contexts. What I meant to say was that LM temperature can be used in order to make model behavior consistent across repetitions of the same input. When giving examples of case counterfactuals, however, I was thinking of the patient's body temperature as a potential biomarker to change. The LM temperature parameter should not be used as a counterfactual.

We thank the reviewer for their clarification on the temperature. We added further analyses into the counterfactual changes to Supplementary Materials 3 and 4 under the section "Body Temperature". The previous conclusions of this preliminary evaluation did not change. Therefore, we made no additional changes to the manuscript.
